# The role of services content for manufacturing competitiveness: A network analysis

**DOI:** 10.1371/journal.pone.0226411

**Published:** 2020-01-14

**Authors:** Leticia Blázquez, Carmen Díaz-Mora, Belén González-Díaz

**Affiliations:** University of Castilla-La Mancha, Cobertizo San Pedro Mártir s/n, Toledo, Spain; Politecnico di Milano, ITALY

## Abstract

This paper explores the phenomenon of international servicification of manufacturing from the period 1995 to 2011. By applying empirical techniques of Social Network Analysis and graph theory, we find that the network of flows of intermediate services embodied in manufacturing exports is still slightly dense and would not correspond to a traditional centre-periphery structure. The mapping shows a numerous, highly cohesive group of countries, with China, the USA and Germany as central economies and an increasing leading role of Asian economies, which would indicate their commitment to upgrading within global value chains. We go a step further by empirically analysing the impact of the countries’ centrality in the global network of intermediate services on manufacturing competitiveness. Our findings reveal that, together with the level of embodiment of intermediate services into manufacturing exports, who the providers of those services inputs are is a key determining factor for manufacturing competitiveness.

## 1. Introduction

The growing importance of services in international trade is widely supported by the empirical literature. The phenomenon becomes even more evident when trade flows are measured in terms of value added. Services account for around 50 percent of world exports according to the OECD-WTO Trade in Value Added (TiVA) database. One of the possible explanations for this expansion could be the ever more segmented and geographically dispersed production processes, as long as efficient and high-quality services are key elements for the proper functioning of these global value chains (GVCs). At the same time, advances in information and communication technologies and fewer obstacles to their international exchanges have enabled services to be more tradable. The whole is a virtuous circle which leads not only to the cheapening and upgrading of these services but also to driving the international fragmentation of production forward.

This growing dependence on services is most clearly addressed in the manufacturing industry [1–5]. In an increasingly competitive and complex environment, manufacturing companies require the provision of specialised services that enable higher operational flexibility, cost savings, product differentiation, and/or search for greater efficiency. One of the consequences of these closer links is the progressive incorporation of foreign value added from the services sector in manufacturing goods, mainly in those destined for export. These services are often produced globally by specialised companies, becoming a crucial element of cross-border manufacturing processes. Moreover, the denser and more complex these GVCs are, the higher the sophistication and quality and, hence, the specialisation of services involved. In this way, access to these services emerges as a requirement for the participation and upgrading of a country in GVCs [6, 7].

Those interdependent dynamics are configuring a world inter-sectoral network in which services are playing an ever more prominent role. Also, the level, intensity and function of countries which participate in the network are not trivial. The role of different countries as demanders or suppliers of intermediate services embodied in manufacturing will largely depend on their comparative advantages, which, in turn, will determine their type of GVC participation. Countries with low labour costs often integrate into GVCs by focusing on manufacturing and assembly phases. For that, they require access to high-quality, highly specialised services in order to maintain and improve their position in the network. In the absence of domestic supply, these services are generally demanded abroad. Therefore, in parallel to GVCs in manufacturing sectors, a global network of suppliers and users of intermediate services to be incorporated into manufacturing has emerged.

The aim of this paper is twofold. The first is to identify the “expert suppliers” within the global network formed by cross-border exchanges of intermediate services embodied in manufacturing exports, and the evolution of the network over time. To do this analysis, we will use data from TiVA database and will apply Social Network Analysis (SNA, hereafter) tools and graph theory to this world network. Secondly, based on the results obtained in the previous analysis, we will go a step further by empirically studying how countries’ expertise in the global network of embodied intermediate services affects manufacturing competitiveness. Our hypothesis is that, together with the level of embodiment of those services, who the providers of those intermediate inputs embodied in manufacturing exports are matters: the higher the performance of services suppliers, the more intense the competitiveness-enhancing effect.

The second part of the analysis is the main contribution to the existing literature. While the effect of using foreign material inputs on manufacturing competitiveness has been widely investigated in recent empirical literature [8–14], the influence of services content of manufacturing exports has scarcely been explored. Among the few exceptions are the works by Francois and Woerz [15] and Wolfmayr [16] for OECD countries, Landesmann and Leitner [17] for EU-27 and Kowalski et al. [7] for a group of developing countries. Most of them focusses on business services. These works find a positive and significant effect of services content on manufacturing competitiveness, which is limited to foreign services according to Wolfmayr [16] results and limited to business services and the most skill and technological-intensive manufacturing industries according to Francois and Woerz [15]. For Landesmann and Leitner [17], the positive impact on manufacturing competitiveness is found for both domestic and foreign intermediate business services. Díaz-Mora et al. [18] study the impact of foreign services value added embodied in manufacturing exports on export duration for a sample of 63 OECD and non-OECD countries in the period 1995–2014. Their findings show a positive effect that is more pronounced for developing and emerging economies and helps export relationships to be more resilient. Liu et al. [19] find a positive influence of the development of financial and business services on manufacturing export competitiveness, but exclusively for those industries that use these services intensively. Furthermore, countries with underdeveloped services may partially overcome that handicap by increasing the foreign services content of their manufacturing sectors. The above-cited papers focus on the quantity and the type of services content of manufacturing exports, but none of them explore the influence of intermediate service country-providers’ characteristics on manufacturing competitiveness. As far as we know, this is the first work that addresses this question.

The article is structured as follows. After this introduction, the second section explains the data and indicators used in the analysis of the characteristics of the network and the role of the countries that form it. This analysis is carried out in Section 3. Section 4 presents the empirical model to test the role of embodied intermediate services in manufacturing competitiveness and discusses the results. Final considerations are reported in Section 5.

## 2. Data and indicators

As mentioned in the Introduction, our main goal is to test whether or not, together with the level of embodiment of services inputs into manufacturing exports, a higher performance of services suppliers has a competitiveness-enhancing effect on manufacturing exports. Data to construct those explanatory variables as well as the dependent variable (manufacturing competitiveness) come from the TiVA database, December 2016 edition. It offers information for the period 1995–2011 for 64 countries (see Table A.1 in S1 Appendix).

### 2.1. Measuring manufacturing competitiveness

Manufacturing competitiveness is measured by a modified version of revealed comparative advantage (RCA). The traditional RCA is the share of a country-sector’s gross exports in the country’s total gross exports, relative to the share of that sector in world gross exports. Instead, the modified RCA indicator takes into account both domestic and international production fragmentation and is based on domestic value added (DVA) embodied in gross exports [20, 21]. Specifically, DVA is the forward linkage-based measure of value added originated in a country-sector and embedded into all the sectors’ gross exports of that country. That is, it is a measure of value added from a producer’s perspective as long as it considers both the domestic value added of a sector exported directly from itself and that exported indirectly when it is embodied as intermediate inputs in other sectors’ exports. The modified RCA index based on DVA for country *i*’s manufacturing sector at time *t* (*RCA*^*M*^_*DVAit*_ for short) is calculated as follows:

$${RCA}_{DVAit}^{M}=\frac{\left(\frac{{DVA}^{M}it}{{DVA}^{Total}it}\right)}{(\sum_{i}{DVA}^{M}it/\sum_{i}{DVA}^{Total}it)}$$ (1)

where *DVA*^*M*^_*it*_ denotes the forward linkage-based measure of value added that originates from the manufacturing sector of country *i* and is embedded in the gross exports from all sectors of that country *i*, at time *t*. Then, *RCA*^*M*^_*DVAit*_ is the ratio between the share of domestic manufacturing value added embodied in country *i*’s gross exports in the country’s total domestic value added embodied in gross exports at time *t*, and the corresponding share for the world. As in the traditional RCA, an *RCA*^*M*^_*DVAit*_ index greater than one would indicate that the country has a revealed comparative advantage in that sector. Conversely, a value lower than one means a revealed comparative disadvantage for that country-sector.

**Fig 1:**
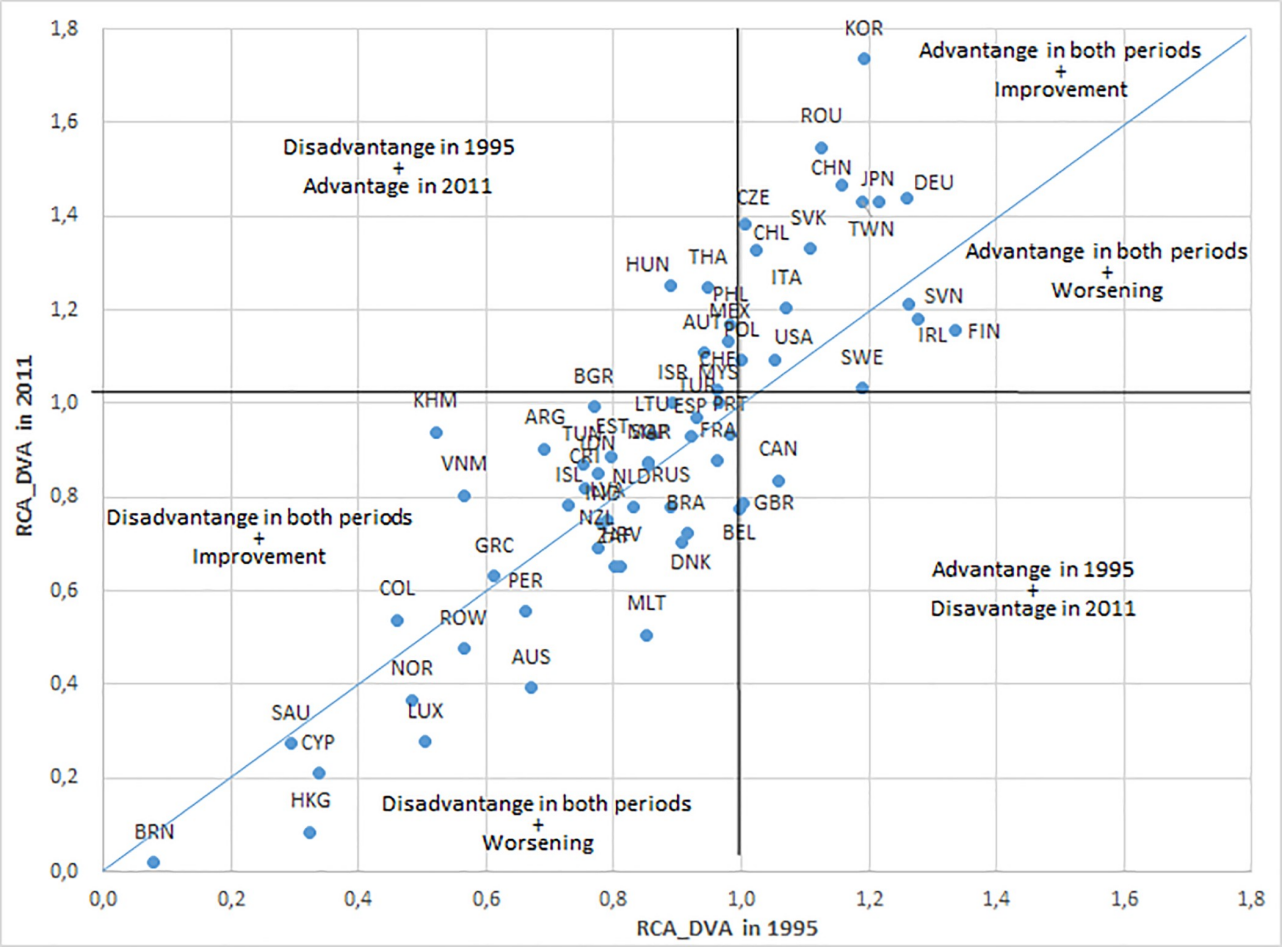
RCA index based on Domestic Value Added for the manufacturing sector, by country, 1995 and 2011. Source: Authors’ elaboration based on OECD-OMC TiVA Database (December 2016).

Fig 1 plots for each country the value of *RCA*^*M*^_*DVA*_ in 1995 and 2011. It is divided into four quadrants. There are more countries located in the bottom left quadrant, where comparative disadvantage prevails in both periods, than in the upper right quadrant, where comparative advantage exists in both periods. This is an expected result since most countries in the sample are high or upper-middle income level economies, with specialisation in the service sector. Additionally, countries located along the diagonal line are those with improvements in manufacturing competitiveness between 1995 and 2011, while those below the diagonal line have seen their manufacturing competitiveness decline. The number of countries in each area is quite similar. The farther the country is from the main diagonal, the greater the change. Advanced countries such as Finland, Ireland, Sweden, Canada, the United Kingdom, Belgium, Denmark, Austria, Luxemburg and Hong Kong exhibit a substantial worsening in manufacturing competitiveness. The first three countries still show comparative advantage in 2011, while Canada has lost it. On the other hand, other advanced countries such as Germany, Japan and Korea show the largest improvements in manufacturing competitiveness. Finally, emerging Asian countries such as China, Thailand, Cambodia and Vietnam and Eastern European countries such as Romania, Slovakia, the Czech Republic, Hungary and Bulgaria have also significantly strengthened their comparative advantage over time.

### 2.2. Measuring expert suppliers of services value added embodied in manufacturing exports

To measure the expertise of suppliers of intermediate services embodied in manufacturing exports, we apply SNA methodology to data on foreign value added which comes from services sectors and is incorporated into manufacturing exports. This information comes from the TiVA database and is calculated using OECD Inter-Country Input-Output (ICIO) tables. Specifically, it is based on the Leontief inverse matrix to capture those final foreign value-added flows in exports after all stages of production have propagated through the world [22]. In this paper, we denote it as *FSVA*_*o*_*inX*^*M*^_*i*_, where *o* is the origin or source country of the foreign services value added (*FSVA*) and *i* is the destination country whose manufacturing exports (*X*^*M*^_*i*_) embody that foreign services value added content.

Since it is reasonable to expect a varying impact of different intermediate services on the competitiveness of manufacturing exports, in addition to aggregate services, we consider four types of services separately in our analysis, according to the ISIC classification: Trade and distribution services (ISIC Rev. 3.1. codes 50–55); Transport and communication services (ISIC codes 60–64); Financial services (ISIC codes 65–67) and Computer, R&D and other business services (ISIC codes 72–74).

SNA methodology is based on mathematical graph theory and thus we must start with the construction of a network with nodes and edges. In our analysis, the nodes are the 64 economies included in the TiVA database, and the edges are the countries’ shares of foreign services value added embodied in their manufacturing exports (FSVA_o_inX^M^_i_) in world services value added embodied in world manufacturing exports values (∑_o_ ∑_i_ FSVA_o_inX^M^_i_). Those shares are denoted by *w*_*oi*_ and measured as:

$$w_{oi}=\frac{\left({FSVA}_{0}{inX}_{i}^{M}\right)}{(\sum_{o}\sum_{i}{FSVA}_{0}{inX}_{i}^{M}〗}$$ (2)

As we are interested in comparing the network at two different moments in time, by using these shares, trend effects are eliminated and we obtain adimensional weights that are automatically deflated, allowing for consistent comparisons across different years and country types [23]. Additionally, in order to consider only relevant flows between countries of services valued added embodied in manufacturing exports, a threshold is set. Specifically, we include only those *w*_*oi*_ larger than 0.03. By imposing this threshold, we cover 85 percent of the total services value added exchanged between the countries included in the sample. The main results obtained are not altered substantially if a slightly lower (or higher) threshold is set.

While we are interested in evaluating the network from both a qualitative and quantitative point of view, we consider it from two complementary standpoints. First, we build a directed weighted network where each directed link is *w*_*oi*_. Additionally, we also explore the properties of the binary projection of the weighted generic matrix (*W*) by analysing the mere presence or absence of a flow of services value added embodied in manufacturing exports between two countries. The elements of this binary matrix (*A*) will be: *a*_*oi*_ = 1, when *w*_*oi*_>0; and *a*_*oi*_ = 0, when *w*_*oi*_ = 0.

For each year, we obtain an input-output table which records those *w*_*oi*_ for any pair of connected economies. As an illustration, Table 1 shows an example for a network with three economies. In this network, the element *w*_*oi*_ with a value of 0.33 (first row, second column) would correspond to the services value added from source country 1 embodied in manufacturing exports of destination country 2 (whose value is 3,300) divided by world services value added embodied in world manufacturing exports (whose value is 10,000). We would have to construct four separate data tables (similar to Table 1), one for each of the four types of services considered in the analysis.

**Table 1:** A hypothetical table for a three-economies network.

Data on *w*_*oj*_			Destination industry: Manufacturing			
			Destination country (country *i*, column country)			
			Country 1	Country 2	Country 3	Total
Source industry: Services	Origin or source country (country *o*, row country)	Country 1	0	3,300/10,000 = 0.33	900/10,000 = 0.09	4,200/10,000 = 0.42
		Country 2	2,000/10,000 = 0.2	0	700/10,000 = 0.07	2,700/10,000 = 0.27
		Country 3	2,500/10,000 = 0.25	600/10,000 = 0.06	0	3,100/10,000 = 0.31
		Total	4,500/10,000 = 0.45	3,900/10,000 = 0.39	1,600/10,000 = 0.16	10,000/10,000 = 1

As long as the network is directed, we can consider the flows of embodied services valued added from two different perspectives: outgoing edges or forward links, and incoming edges or backward links. The first ones (row links in Table 1) identify the clients of each source country, i.e., those countries which embody services valued added that come from the source country into their manufacturing exports. In the SNA nomenclature, the number of those destination countries will be the outdegree of each node (∑_*i*_*a*_*oi*_), and the total amount of services value added that each country provides to all destination countries to be embodied in their manufacturing exports (as a percentage of the world total) will be its outstrength (∑_*i*_*w*_*oi*_). Conversely, the backward links (column links in Table 1) identify the providers of foreign services value added of each manufacturing exporting country. The number of those provider countries will be the indegree of each node in the SNA terminology (∑_*o*_*a*_*oi*_), and the total amount of foreign services value added that each manufacturing exporting country embodies from all its provider countries (as a percentage of the world total) will be its instrength (∑_*o*_*w*_*oi*_).

As the increasing role of those embodied intermediate services has resulted in changes in the network structure (and vice versa), SNA is an appropriate methodology since it enables us to measure the nature and evolution of these intermediate services-manufacturing relations between countries in ways that other measurements do not capture. These changes comprise a double dimension: (i) How many, which, and how countries participate in the network; and (ii) With what intensity, in terms of volume of embodied services value added, they will do it. By analysing the changes in these dynamics and identifying those countries that have played a prominent role in them, we will be able to detect the expertise suppliers of embodied services value added within the network.

Of course, the influence of a node in the network is largely affected and reflected by the topological structure of the network it belongs to, and the interpretation of what is meant by “important” in a network depends on what the aim of the analysis is. Accordingly, many different measures of node-centrality have been developed. All of them assign a value to each node ranking the nodes subject to their importance. Roughly, three kinds of node-centrality indicators have been developed in the related literature [24]: (i) centrality measures essentially based on neighbourhood, such as degree of nodes, i.e., the number of partners with which each economy has established direct exchanges of embodied services value added, strength, in the case of the weighted network, or clustering coefficients; (ii) measures based on distance or path-based centralities, such as closeness centrality, i.e., the geodesic distances between countries, or random-walk betweenness centrality, which counts the expected times a node is traversed in a random walk between an arbitrary pair of nodes, considering all paths in the network and giving more weight to shorter paths, i.e., considering the importance of countries as intermediaries; and (iii) measures not only based on the number of neighbours or the weight of the links that nodes establish with them, but also determined by their influence, i.e., considering the *mutual enhancement effect*. Examples of these third iterative refinement centralities in which every node gets support from its neighbours are eigenvector centrality and cumulative nomination algorithm for undirected networks, and page rank and *Hyperlink-Induced Topic Search* (HITS) algorithms (and their variants) for directed networks.

The HITS algorithm was originally introduced by Kleinberg [25] to rate the importance of a node in a complex directed network considering the different roles played by nodes: *authorities* and *hubs*. In its original meaning, a node with a high authority value is indicated by many other nodes with high hub values, and a node with a high hub value indicates many nodes with high authority values. In a directed network, the authority score of a node equals the summation of the hub scores of all the nodes that point to this node while the hub score of a node equals the summation of the authority scores of all the nodes which this node points to. As is well known, in the PageRank algorithm the importance of a node is determined only by the importance of the nodes pointing to it, i.e., as an *authority*. Thus, in the case of our network, the weighted HITS is more nontrivial, as it reflects the two-links network structure [26]. Although the Reverse PageRank algorithm has also been developed to calculate the importance of nodes as hubs, its effectiveness on this measure is still not completely clear [27].

Since we are interested in identifying those high-quality, highly specialised services providers within the network, i.e., those countries considered by their partners to be “expert suppliers”, in this paper we will specifically apply the weighted HITS algorithm, that is, the quantitative measures of *hubs* and *authorities*, to clarify the network topology changes more exactly in terms of network science than the original HITS does [26, 28]. Although these qualitative network measures are correlated with countries’ economic sizes, that is, they take large values for large countries, their values also depend on their network location. Consequently, in our network, the *hubs* will be those economies that not only supply services value added to other economies which do not produce them domestically, but also are the origin of a very intense flow from them towards other countries which are sophisticated recipients themselves, central countries within the network as suppliers, i.e., the more discerning customers that choose the best suppliers.

## 3. The expert suppliers: Mapping out the world embodied intermediate services network

In SNA, the within-sample dynamics of the network and its main characteristics can be summarised by topological measures and paths. Before identifying who the expert suppliers in the embodied services value added network are, it is necessary to contextualise these measures, i.e., to provide evidence for the structural properties of the whole network and its evolution: the number of partners and the interaction intensity of countries (its density); the volume of embodied services value added exchanged; the shape of the network, more centred or more regular; the heterogeneity of the role of its members as intermediaries in the network; and whether or not and to what extent there exist relatively dense subnetworks and thus cohesive subgroups within the whole network. These measures can consider both first-order relations between countries, i.e., direct link with their partners and second-order relations, which indicate how and how much each country’s partners are themselves connected within the network.

### 3.1. Network structure

**Fig 2:**
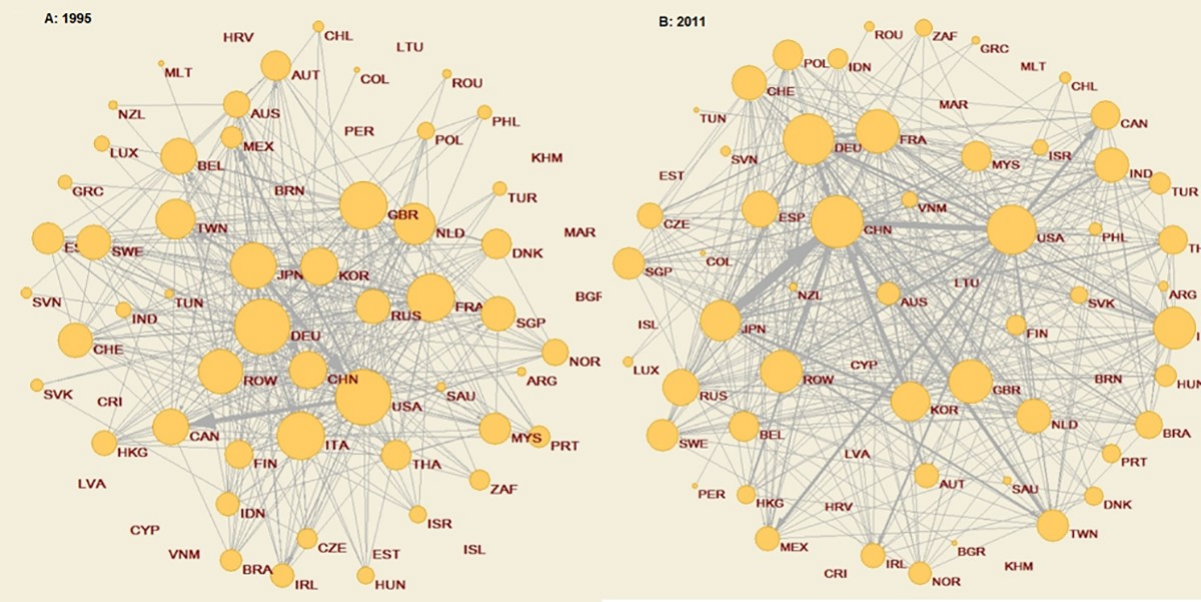
Network evolution, 1995 and 2011. Note: The size of the nodes (countries) is related to their total number of links (*all node degrees*). The links between countries reflect the flows of intermediate services embodied in manufacturing exports. Source: Authors’ calculation based on OECD-OMC TiVA Database (December 2016) using the program package Pajek for analysis and visualisation of large networks (http://mrvar.fdv.uni-lj.si/pajek/).

We start the analysis by studying the evolution of aggregate statistics of the binary network, specifically, the **first-order indicators**, from 1995–2011. Fig 2A and 2B and Table A.2 in S1 Appendix reveal that, for the period of study, non-significant structural changes were detected in the network. The network has become slightly denser, although its current connectivity could be considered incipient. To some extent, this is an expected result, in line with previous literature. It seems quite reasonable to believe that transnational networks are less developed and integrated when we consider services instead of goods [22]: service fragmentation has taken place, sequentially, later than physical production fragmentation. We also observe that the network is moderately more extensive in 2011 than in 1995: countries have increased their *average node degree*. Considering the binary network from a double perspective, suppliers and recipients, we observe that the differences between both networks are not significant.

According to the results for aggregate centrality measures (Table A.2 in S1 Appendix), the network would not respond to a traditional centre-periphery structure in terms of connectivity and intensity. Firstly, the *degree centralisation* indexes, i.e., the extent to which a network is centred on one or several important nodes, are fairly moderate and decreasing, which would indicate that the network exhibits a poorly integrated structure. However, these results (and, in general, all the aggregate centrality indexes) must be considered with certain caution, as they can be influenced by the low number of countries included in the sample (64 countries). Moreover, most of those countries play a leading role in the international economy. In Fig 2A, we appreciate at the beginning of the period a numerous group of highly connected countries in the centre with trade relationships with many other countries. Additionally, we also observe that new countries have been incorporated into this centre over time, playing a prominent role. The most evident example is China. This increasingly regular structure of the network might imply a lower probability that asymmetric shocks that affect specific nodes quickly spread to the rest of the economies through cascading effects. When the network is analysed from the double perspective of suppliers and recipients, the results of the *outdegree* and the *indegree centralisation indexes* are low. However, it is remarkable that while providers increasingly diversify, the network of recipients becomes more concentrated over time. The rest of the indexes calculated ratify the irregular shape of the network. The low average *random walk betweenness centrality* (RWBC) index would signal the small number of countries with a crucial role as intermediaries in the network in both periods of analysis. It also implies a certain symmetry in the relationship of countries since their function as gatekeepers in the network is quite homogeneous. Nevertheless, according to the *closeness centrality index*, the geodesic distances between countries seem to have shrunk, and, in both years, more links seem to imply more direct links, which would suggest that the central countries within the network will be the ones that bolster their integration. The *clustering coefficient* is relatively high, and basically constant over time.

**Fig 3:**
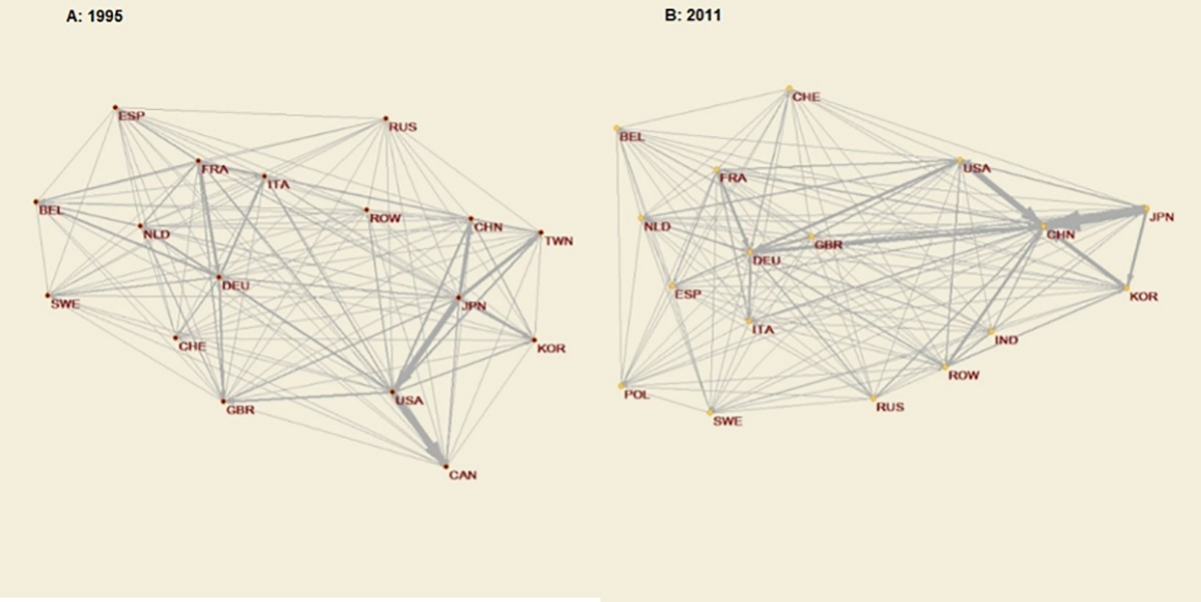
*K-Cores* in the network, 1995 and 2011. Source: Authors’ calculation based on OECD-OMC TiVA Database (December 2016) using the program package Pajek for analysis and visualisation of large networks (http://mrvar.fdv.uni-lj.si/pajek/).

In order to identify clusters of countries that are closely connected, we apply the so-called *k-cores* analysis (Fig 3). In this way, we can detect the maximal subnetwork in which each node has at least degree *k*. It therefore identifies relatively dense subnetworks and thus cohesive subgroups within the whole network. In 1995, the densest *k-core*, which contained nodes with at least a degree of 22, was made up of 17 countries. In 2011, the same number of countries composed the densest *k-core*, in this case with at least a degree of 23. Consequently, we have, within this network, a wide subnetwork with countries strongly connected with each other. They are, indeed, the crucial actors within the network over time. In 2011, they were EU members such as Belgium, France, Germany, Italy, the Netherlands, Poland, Spain, Sweden and the United Kingdom; other European countries like Switzerland and Russia; Asian countries like Japan, Korea, China and India; and the United States. Canada and Taiwan were also part of the densest connected subnetwork in 1995, while Poland was part of it only in 2011, after its incorporation into the UE in 2004 and hand in hand with German transnational production fragmentation processes.

When we go a step further and consider the intensity of flows of the intermediate services (weighted network), we observe that average contribution that exchanges maintained by countries with their partners to the world trade of embodied services inputs (average *strength*) has remained almost unaltered over time. By interacting the binary and the weighted network, we have detected that the most connected and integrated countries are also the most intensely connected. Consequently, the role of these countries in the future development of the network is reinforced.

When the weighted network distinguishes recipients (*instrength*) and suppliers (*outstrength*), we observe different although convergent dynamics between them over time. While in 1995 the importers’ intermediate services network was more homogenous, with countries contributing in a more symmetric way to the world added value, in 2011 some countries concentrated foreign intermediate services demand to a greater extent. In particular, in 1995, the United States was the country with the highest *instrength*, with 8.2; in 2011, China was the first country in the ranking, with a share of 16.5, practically double. From the supply side, the dynamic is fairly different. In 1995, there were fewer important providers than recipients, with a more concentrated contribution. However, in 2011, we observe a more diversified supply. These results would suggest that some economies are boosting the international terciarisation of manufactures in a firmer way than others. The weighted centrality measures reinforced the results of the binary analysis. Nevertheless, considering both, the increasing tendency of these indexes and the results obtained when we correlate the *betweenness* binary indexes (RWBC) with the *strengths* of countries would indicate that those economies with the most intense links would presumably be the ones that will gain a more central role as intermediaries within the network. Consequently, the performance of those leading countries (in terms of volume or share) related to their productive, legal, organisational and property development of intermediate services will be crucial for the future evolution of this world network.

Finally, when we consider the **second-order indicators**, we observe that the average number of partners that every country’s partners have themselves (*average nearest-neighbour degree*, ANND) is much higher than the *average node degree* in both periods, and that these differences have increased. Therefore, we infer that the central countries within the network are extending their links towards other poorly connected economies. Additionally, the correlation between *node strength* and *average nearest-neighbour strength* (ANNS) is strongly negative in both periods, indicating that only one group of countries has intensified their flows, enhancing their leading role within the network.

As we mentioned in the Introduction, it is interesting to analyse the performance of the network for the different types of services. When we do that, we observe that all of them show structural characteristics similar to those of the total network: very moderate connectivity, with *Transport and communication services* the densest section in both periods and *Computer*, *R&D and other business services* the least connected. Similarly, all four sectional networks are modestly extensive, and all of them have increased their *average node degree*: the highest *average degree* is shown by *Transport and communication services* and the lowest by *Computer*, *R&D and other business services* in both periods, but all of them are analogous to the average of the total network.

Besides, as for the total network, all centralisation coefficients would indicate regular networks for the four sections. The *degree centralisation index* is moderate in all sectional networks. In 1995, the figures were even lower for the sectional networks than for the total network, while in 2011, the results were more aligned. The most centred sectional network is that of *Financial services*. It is also interesting to observe that only in the *Transport and communication services* section has the number of direct links between nodes increased, as we have seen for the total services network.

Considering the weighted network, in both periods, the *average strength* was higher for all sectional networks than for the total network, and was especially higher for *Trade and distribution services* in 2011. In fact, the *average strength* has increased noticeably in this section, which would indicate that the average contribution of countries to the world trade of these intermediate services has grown. We also observe a higher concentration of supply, with the five principal economies (excluding ROW) concentrating half of the world share in 2011 (40 percent in 1995). The contrary situation is depicted in the other three sections, especially in *Computer*, *R&D and other business services*, for which the average contribution of economies has decreased over time and the concentration has diminished.

### 3.2. Who are the hubs in the network?

We examine in more detail the composition of the network centre by analysing the node-specific measures, focusing on the analysis of the results of the weighted HITS algorithm. Firstly, it is important to note that, in both periods, out of the top 20 countries considered *hubs*, 14 were also *authorities* (15 if we include the rest of the world). It is likely that transnational companies’ operations from these countries are behind this shared leading role from the demand and supply perspective.

Table 2 displays the ranking and evolution of hubs in the 1995–2011 period. We can observe that, in 2011, the top positions in the ranking of hubs in the network are occupied by some Asian countries, with China at the head together with Korea (position 3), Taiwan (5), Malaysia (11), Thailand (13) and Japan (14); European countries, with the central EU economies as leaders, particularly with Germany at the forefront (2), but also France (8), Italy (9), the United Kingdom (10), Ireland (12) and the Netherlands (15); and North American countries, with the USA (4) as the principal economy and Canada (6) and Mexico (8) also ranking among the top ten. Therefore, in 2011, we can conclude that, similar to the configuration of the physical or good production GVCs, three relevant centres can be detected within the network, with a regional scope and connected themselves: the European Factory, the North American Factory, and the Asian Factory [29]. This intense connection allows the network to achieve a global dimension. One possible explanation for this similarity is that these intermediate services are, in turn, embodied in the intermediate goods throughout the different phases of the value chain, given that they are essential to participating in GVCs in a solid and stable manner. It is very interesting to observe that, in 2011, the majority of these hubs are also the 20 most central countries (with the highest *node degree*), the most intensely connected economies (with the highest *node strength*), the most easily reachable (with the highest closeness centrality) and with the most prominent role as intermediaries (*RWWC*). However, significant changes can be appreciated in some of them with respect to their position.

**Table 2:** Ranking and evolution of *hubs*, 1995–2011.

	Total Services				Trade and distribution services				Transport and communication services				Financial services				Computer, R&D and other business services			
Rank	Country	Value in 2011	Multiplication factor 1995–2011	Change Rank (1995–2011)	Country	Value in 2011	Multiplication factor 1995–2011	Change Rank (1995–2011)	Country	Value in 2011	Multiplication factor 1995–2011	Change Rank (1995–2011)	Country	Value in 2011	Multiplication factor1995-2011	Change Rank (1995–2011)	Country	Value in 2011	Multiplication factor 1995–2011	Change Rank (1995–2011)
1	CHN	0,789	2,83	3	CHN	0,845	2,53	2	CHN	0,834	2,28	1	CHN	0,873	2,79	2	CHN	0,550	4,408	15
2	DEU	0,279	0,91	1	KOR	0,267	1,08	3	KOR	0,248	1,25	7	DEU	0,218	0,61	0	DEU	0,454	1,279	0
3	KOR	0,244	1,20	5	TWN	0,191	0,52	-1	DEU	0,233	0,71	1	KOR	0,151	0,83	9	IRL	0,299	0,971	1
4	USA	0,166	0,45	-2	USA	0,189	0,33	-3	TWN	0,175	0,66	1	USA	0,140	0,47	0	KOR	0,219	1,654	10
5	TWN	0,164	0,61	0	DEU	0,184	0,80	1	USA	0,142	0,43	-2	CAN	0,127	0,29	-4	FRA	0,218	0,723	0
6	CAN	0,151	0,37	-5	MEX	0,133	0,80	3	CAN	0,119	0,31	-5	TWN	0,115	0,55	2	CAN	0,198	0,517	-5
7	MEX	0,149	0,74	3	CAN	0,114	0,39	-3	MYS	0,113	0,81	7	IRL	0,111	0,95	9	NLD	0,175	0,568	-4
8	FRA	0,136	0,56	-1	THA	0,113	1,02	4	THA	0,110	1,14	11	MEX	0,105	0,54	3	MEX	0,175	0,965	2
9	ITA	0,120	0,63	2	MYS	0,108	0,82	2	SGP	0,108	0,51	-1	FRA	0,102	0,41	-4	GBR	0,174	0,617	-3
10	GBR	0,116	0,47	-4	FRA	0,089	0,55	0	FRA	0,105	0,46	-3	THA	0,101	0,92	7	ITA	0,167	0,674	-3
11	MYS	0,102	0,95	7	ITA	0,083	0,79	2	ITA	0,100	0,51	-1	JPN	0,097	0,63	3	ESP	0,127	0,961	4
12	IRL	0,098	0,66	2	JPN	0,074	1,26	6	MEX	0,099	0,72	3	ITA	0,091	0,46	-2	USA	0,126	0,747	-1
13	THA	0,098	1,09	7	GBR	0,073	0,39	-5	ROW	0,095	0,74	3	IND	0,089	8,74	27	SGP	0,120	0,647	-4
14	JPN	0,093	0,80	1	ROW	0,072	0,85	2	GBR	0,090	0,37	-8	GBR	0,088	0,39	-8	CHE	0,112	1,173	5
15	NLD	0,088	0,47	-3	IND	0,061	20,30	26	JPN	0,086	0,57	-3	SGP	0,087	0,39	-8	TWN	0,107	0,789	-2
16	ROW	0,086	0,78	0	ESP	0,051	0,87	3	IND	0,083	8,90	22	ROW	0,087	0,61	-1	JPN	0,107	0,863	1
17	SGP	0,082	0,41	-8	SGP	0,050	0,24	-10	ESP	0,066	0,70	3	MYS	0,082	0,98	3	SWE	0,091	0,560	-5
18	ESP	0,079	0,81	1	NLD	0,047	0,55	-3	RUS	0,059	1,42	9	NLD	0,061	0,30	-9	BEL	0,089	0,401	-10
19	IND	0,072	13,71	22	RUS	0,039	1,60	8	NLD	0,053	0,37	-6	ESP	0,053	0,53	-1	ROW	0,083	0,949	1
20	CHE	0,063	0,75	1	CZE	0,038	2,91	12	CHE	0,047	0,58	1	RUS	0,050	1,62	6	MYS	0,082	1,546	2
21	CZE	0,053	2,56	12	POL	0,034	8,08	17	BEL	0,044	0,24	-10	CHE	0,039	0,54	1	POL	0,075	4,228	13
22	RUS	0,051	1,54	4	CHE	0,032	0,65	0	POL	0,040	5,17	19	BEL	0,039	0,25	-9	CZE	0,071	2,605	6
23	POL	0,051	3,71	12	VNM	0,029	5,16	12	CZE	0,040	2,25	11	CZE	0,035	1,92	11	IND	0,067	11,568	19
24	BEL	0,049	0,29	-11	HUN	0,026	5,36	13	SWE	0,038	0,33	-7	TUR	0,031	6,48	21	HUN	0,064	1,697	1
25	SWE	0,048	0,45	-8	TUR	0,026	12,55	20	TUR	0,029	6,74	20	POL	0,031	1,51	8	AUT	0,056	0,573	-7
26	HUN	0,041	2,26	8	SWE	0,025	0,43	-6	IRL	0,029	0,26	-8	SWE	0,030	0,31	-7	THA	0,055	1,223	-2
27	AUT	0,033	0,49	-5	BEL	0,024	0,26	-13	HUN	0,028	2,88	10	HUN	0,027	1,78	10	RUS	0,053	1,952	2
28	TUR	0,027	12,04	20	SVK	0,020	9,79	16	VNM	0,027	2,12	7	AUT	0,022	0,29	-7	FIN	0,048	0,566	-7
29	VNM	0,023	2343,93	22	IRL	0,017	0,23	-12	AUT	0,023	0,46	-5	VNM	0,019	1882,09	22	DNK	0,039	0,820	-6
30	FIN	0,021	0,41	-7	AUT	0,016	0,47	-7	DNK	0,017	0,25	-8	BRA	0,018	1,15	5	TUR	0,028	11,648	17

Source: Authors’ calculation based on OECD-OMC TiVA Database (December 2016)

From 1995 to 2011, we can observe interesting changes in the role that these central countries play within the network. The most remarkable change is the spectacular variation in China’s hub score. It almost tripled since 1995, allowing this economy to rise three positions in the ranking to become the first world intermediate services hub, with a value very far from that of other countries. Among the first positions, also noteworthy is the advance of Korea, which has increased its hub score until occupying the third position, when it ranked eighth in 1995. Also in Asia, we observe that, by maintaining their hub scores, Malaysia and Thailand have risen markedly since 1995. India, in the middle part of the ranking, has also made remarkable improvement, multiplying its score (13.7) to be amongst the top twenty in the ranking (19). Also noteworthy is the progress of some East European countries such as the Czech Republic, Poland, Hungary and Russia, which have managed to position themselves better in the ranking. Finally, it is worth mentioning the cases of Turkey and Vietnam, which have certainly progressed since 1995. On the other hand, the three North American economies have reduced their hub scores, but while Mexico has improved its relative position, Canada and the USA have fallen in the ranking, especially Canada, which dropped five positions. In Europe, Belgium, Sweden, Austria, the United Kingdom and the Netherlands have seen their scores substantially worsen, falling in the classification. In addition, in Asia, Singapore is the economy with the worst evolution, losing eight positions.

When we disaggregate the network of embodied services value added by services sections, we observe in 2011 a very high correlation between the positions that countries occupy in the ranking of hubs: the same countries are at the top of the list of hubs in all of the four sections. The lowest correlation in the ranking is observed between the *Computer*, *R&D and other business services* section and the other three. However, we do not observe the same patterns when we consider the evolution of hub scores in the different sections, as they are not significantly correlated, which would indicate that differentiated dynamics can be observed in each of the four sectional networks.

Going further in the analysis of these sectional networks, we observe that for the *Trade and distribution services*, *Transport and communication services* and *Financial services* sections, among the top five are China, Korea, the USA and Germany, with great distance between China’s hub score and the rest of the countries’. However, in the *Computer*, *R&D and other business services* section, China, Germany and Korea are also among the top five, but not the USA: Ireland and France occupy the third and fifth positions, respectively. Another significant difference with the other three networks is that China’s hub score in this section network is much closer to the rest of the countries’ and it is lower. Therefore, it is a more diversified network, less concentrated than the other three. In this section, we observe in the top ten positions countries that are not present in the other networks, such as the Netherlands, the United Kingdom and Italy.

As we have mentioned above, the evolution of the four networks has been relatively uneven. China is the country which presents a better evolution in its performance in the four sectional networks: it more than doubles its score in the *Trade and distribution services*, *Transport and communication services* and *Financial services* sections, and it multiplies by more than four its score in the *Computer*, *R&D and other business services* section. Korea improves its scores in all sections but *Financial services*, although it rises in position in all four networks. Thailand increases its scores in *Trade and distribution services*, *Transport and communication services* and *Computer*, *R&D and other business services*, achieving better positions only in the two first; however, it rises in *Financial services* even though its score is worse than in 1995. Japan increases its hub score slightly in *Trade and distribution services*, rising six positions, and rises in *Financial services* in spite of recording a lower score in 2011. As for the total services network, India and Turkey are the countries which present the strongest advance, multiplying their scores intensely in each of the four sections, especially in *Trade and distribution services* and *Computer*, *R&D and other business services*. These improvements mean significant ascents in the rankings. As in the total network, we observe in the four sections notable improvements in some Eastern European countries (the Czech Republic, Poland and Hungary) and, to a lesser extent, Russia, which jump in the rankings. However, the progress is especially significant in the *Trade and distribution services* section for Poland and Hungary, while in the *Transport and communication services* and *Computer*, *R&D and other business services* sections, Poland records the most important advances by far. Also remarkable are the improvement of Switzerland in the *Computer*, *R&D and other business services* section in both score and position and the advance of Spain which maintained its score.

By contrast, we observe that the majority of central European economies have reduced their hub scores substantially over time in the four sectional networks, and some of them have seen their position in the ranking of expert providers drop sharply. That is the case of Belgium, Sweden, Austria, the Netherlands and the United Kingdom. Italy and France have reduced their scores in all sections but Italy has maintained and even improved its position in *Trade and distribution services*, and France in *Computer*, *R&D and other business services*. Other European economies have also reduced their scores and lost positions in some specific sections. For example, Ireland has reduced its scores dramatically in the *Trade and distribution services* and *Transport and communication services* sections; Denmark in *Transport and communication services* and *Computer*, *R&D and other business services*; and Finland in *Computer*, *R&D and other business services*. Noteworthy is the case of some other European economies, such as Germany, Spain and Switzerland, which in this highly competitive context have reduced their scores in the majority of sections, but have managed to maintain their positions in the ranking or even improved them slightly.

Beyond Europe, we observe other interesting dynamics. For instance, the USA has reduced its hub scores in the four sections, but in *Financial services* has maintained its position and in *Computer*, *R&D and other business services* has fallen only one position. Canada, on the contrary, has lost positions in all sections. In this area, Mexico is the only economy, which has improved its positions in the four sections, in spite of reducing its scores in all of them but *Computer*, *R&D and other business services*. Finally, in Asia, Singapore is the economy, which has lost positions in the four sections, especially in *Trade and distribution services* and *Financial services*. Japan has fallen only in *Transport and communication services*, although it has reduced its scores in *Financial services* and *Computer*, *R&D and other business services*.

All in all, we have observed that some emerging economies, especially Asian and Eastern European economies, have gained momentum over time as *hubs* in the network, while some of the most advanced economies in Europe and North America have lost impulse in playing this role under the pressure of those new actors. All sectional networks have incorporated to a greater or lesser extent new countries which are moving up the ranking of expert intermediate services suppliers.

## 4. Empirical model

Once we have examined the structural configuration of the network of embodied services value added and detected the central countries within it, we go a step further by empirically analysing the impact of this centrality on manufacturing competitiveness. The list and definition of variables included in the model are provided in Table A.3 in S1 Appendix.

As we explained in the Introduction, a variable that should positively affect the *RCA*^*M*^_*DVA*_ of a country *i* in a year *t* is foreign services value added embodied in country *i*’s gross manufacturing exports in that year (*FSVA*_*o*_*inX*^*M*^_*it*_) as a share of those gross manufacturing exports. We have seen how the literature has shown that, in addition to being key enablers of GVCs, intermediate services may enhance manufacturing competitiveness by raising productivity, reducing costs and contributing to product differentiation. As is shown in Table A.4 in S1 Appendix, which displays some descriptive statistics of the model variables (before taking logarithms), foreign services value added in manufacturing exports accounts, on average, for 15.5 percent of manufacturing exports, with Japan reporting the lowest share (3.11 in 1995) and Luxembourg the highest (40.8 in 2010). Moreover, the country average share of foreign services value added content of manufacturing exports increases from 13.0 percent in 1995 to 16.4 percent in 2011.

It seems reasonable to consider that, together with the level of embodiment of services value added, the level of expertise of their providers (that is, the level of expertise of the source countries of the embodied services value added) is also relevant: the higher the level of expertise of the embodied services suppliers, the more intense the competitiveness-enhancing effect is. As we observed in Section 3.2, a suitable indicator for proxying the level of a country as a specialised (“expert”) provider is its *hub* score. As we noted, a country is considered a prominent *hub* within the network when it links prominent authorities, i.e., when it supplies intermediate services to countries that are provided by many other prominent suppliers. Therefore, it supplies “expert” buyers. The variable hub ranks in our sample between 0 (Costa Rica, Cyprus) and 0.79 (China). Our hypothesis is that the higher the *hub* score of a country’s suppliers is, the higher its *RCA*^*M*^_*DVA*_ is. Then, we propose to calculate a provider-weighted indicator of foreign services value added content of manufacturing exports (*o_Weighted_FSVA*_*o*_*inX*^*M*^_*it*_), where the share of the embodied services value added from each source or supplier country is weighted by each *hub* score. The average of this weighted indicator is 2.8 (Table A.4 in S1 Appendix). Again Japan and Luxembourg show the lowest (0.65) and the highest (8.5) values, respectively, and an increasing trend is observed between 1995 and 2011.

Furthermore, services value added to be incorporated in manufacturing exports can be supplied by domestic providers and not only by foreign providers. When a country’s domestic providers are highly specialized, that country will play a prominent role as a hub within the network of embodied services value added. It can also be presumed that the country *hub* score itself (*Hub*_*it*_) positively affects the country’s manufacturing competitiveness. Consequently, we should control for it in the model. Moreover, we control for the share of domestic services embodied in a country’s manufacturing exports (*DSVAinX*^*M*^_*it*_). According to the descriptive statistics from Table A.4 in S1 Appendix, the average of this variable (19.2) is higher than that of foreign content (15.5), with Cambodia reporting the lowest share (2.2) and Hong Kong the highest (43.1). Between 1995 and 2011 the average share of domestic services value added in manufacturing exports decreased slightly. Furthermore, we would expect domestic intermediate services content of manufacturing exports of those countries with a high hub score to enhance their manufacturing competitiveness. To capture this effect, we construct a country *i*-weighted indicator of domestic services value added content of manufacturing exports (*i_weighted_DSVAinX*^*M*^_*it*_), where the share of those domestic services is weighted by the exporting country’s *hub* score. With this measure, we try to quantify the combined effect of incorporating more or less domestic intermediate services in manufacturing exports and the role of the exporting country itself as a *hub* on that country’s manufacturing competitiveness. The average of this indicator, which ranges from 0 (Cyprus) to 10.54 (China), is 1.26.

Additional variables, those most commonly used in empirical *RCA* analysis, are included in the model as control variables (*Z*_*it*_ denotes the vector for these control variables). Firstly, we have considered labour productivity in the manufacturing sector in order to account for price and non-price factors of competitiveness. Labour productivity is measured as real value added divided by total employment. We also control for the size of the exporting country’s manufacturing sector, measured by its manufacturing employment, and for the country’s development level, using GDP per capita, both of them in logarithms. We add country fixed effects to control for unobserved time-invariant country characteristics (*a*_*i*_) and time fixed effects to control for unobserved factors that are common to all countries (*a*_*t*_). Robust standard errors are clustered by country to control for potential heteroscedasticity and country-level serial correlation in the error terms.

In line with the aforementioned arguments and the availability of data, we estimate the following specifications:

$${{RCA}^{M}}_{DVA_{it}}=\beta_{0}+\beta_{1}\;{{FSVA}_{o}inX}_{it}^{M}+{a_{i}+a_{t}+\varepsilon }_{it}$$ (3)

$${{RCA}^{M}}_{DVA_{it}}=\beta_{0}+\beta_{1}\;o\_weighted\_{{FSVA}_{o}inX}_{it}^{M}+{a_{i}+a_{t}+\varepsilon }_{it}$$ (4)

$${{RCA}^{M}}_{DVA_{it}}=\beta_{0}+\beta_{1}\;{{FSVA}_{o}inX}_{it}^{M}+Z_{it}+{a_{i}+a_{t}+\varepsilon }_{it}$$ (5)

$${{RCA}^{M}}_{DVA_{it}}=\beta_{0}+\beta_{1}\;o\_weighted\_{{FSVA}_{o}inX}_{it}^{M}+Z_{it}+{a_{i}+a_{t}+\varepsilon }_{it}$$ (6)

$${{RCA}^{M}}_{DVA_{it}}=\beta_{0}+\beta_{1}\;{{FSVA}_{o}inX}_{it}^{M}+\beta_{2}\;{Hub}_{it}+Z_{it}+{a_{i}+a_{t}+\varepsilon }_{it}$$ (7)

$${{RCA}^{M}}_{DVA_{it}}=\beta_{0}+\beta_{1}\;o\_weighted\_{{FSVA}_{o}inX}_{it}^{M}+\beta_{2}\;{Hub}_{it}+Z_{it}+{a_{i}+a_{t}+\varepsilon }_{it}$$ (8)

$${{RCA}^{M}}_{DVA_{it}}=\beta_{0}+\beta_{1}\;{{FSVA}_{o}inX}_{it}^{M}+\beta_{2}\;{Hub}_{it}+\beta_{3}\;{DSVAinX}_{it}^{M}+Z_{it}+{a_{i}+a_{t}+\varepsilon }_{it}$$ (9)

$${{RCA}^{M}}_{DVA_{it}}=\beta_{0}+\beta_{1}\;o\_weighted\_{{FSVA}_{o}inX}_{it}^{M}+\beta_{2}\;{Hub}_{it}+\beta_{3}\;{DSVAinX}_{it}^{M}+Z_{it}+{a_{i}+a_{t}+\varepsilon }_{it}$$ (10)

$${{RCA}^{M}}_{DVA_{it}}=\beta_{0}+\beta_{1}\;{{FSVA}_{o}inX}_{it}^{M}+\beta_{2}\;i\_weighted\_{DSVAinX}_{it}^{M}+Z_{it}+{ai+at+\varepsilon }_{it}$$ (11)

$${{RCA}^{M}}_{DVA_{it}}=\beta_{0}+\beta_{1}\;o\_weighted\_{{FSVA}_{o}inX}_{it}^{M}+\beta_{2}\;i\_weighted\_{DSVAinX}_{it}^{M}+Z_{it}+{ai+at+\varepsilon }_{it}$$ (12)

Table 3 displays the econometric results of the different specifications of the proposed *RCA*^*M*^_*DVA*_ model. In all of them, “services” refers to total aggregate services. In column (1), the variable *FSVA*_*o*_*inX*^*M*^_*i*_ (the share of foreign services valued added content of manufacturing exports) is the only one included (Eq 3). The coefficient is positive but not statistically significant. That is, countries’ revealed comparative advantage in manufacturing does not seem to depend on foreign services value added embodied in their manufacturing exports.

**Table 3:** Empirical model estimation results. Total aggregate services.

VARIABLES	Column 1	Column 2	Column 3	Column 4	Column 5	Column 6	Column 7	Column 8	Column 9	Column 10
**FVAS_o_inX^M^_it_**	0.0690		0.223**		0.133*		0.00368		0.170*	
	(0.0495)		(0.0827)		(0.0772)		(0.0943)		(0.0864)	
**o_weighted_FVAS_o_inX^M^_it_**		0.0699*		0.189**		0.114*		0.0145		0.139*
		(0.0403)		(0.0766)		(0.0658)		(0.0703)		(0.0776)
**Hub_it_**					0.0620**	0.0621**	0.0583***	0.0595**		
					(0.0229)	(0.0231)	(0.0209)	(0.0220)		
**DVASinX^M^_it_**							-0.303***	-0.310***		
							(0.106)	(0.0996)		
***i*_weighted_DVASinX^M^_it_**									0.0463**	0.0457**
									(0.0173)	(0.0177)
**Employment**			0.345***	0.348***	0.267**	0.269**	0.200*	0.197*	0.298***	0.301***
			(0.106)	(0.106)	(0.104)	(0.104)	(0.105)	(0.105)	(0.104)	(0.104)
**Labour Productivity**			0.0884*	0.0807*	0.0401	0.0359	0.0175	0.0163	0.0562	0.0509
			(0.0473)	(0.0459)	(0.0436)	(0.0431)	(0.0513)	(0.0500)	(0.0435)	(0.0429)
**GDPPC**			0.113	0.126	0.0775	0.0816	0.156	0.153	0.0749	0.0830
			(0.108)	(0.113)	(0.107)	(0.112)	(0.125)	(0.127)	(0.112)	(0.116)
**Constant**	0.681***	0.804***	-3.256**	-2.970**	-1.724	-1.514	-1.304	-1.195	-2.191	-3.083*
	(0.0796)	(0.0164)	(1.321)	(1.290)	(1.391)	(1.377)	(1.151)	(1.245)	(1.452)	(1.545)
**Observations**	1,088	1,088	578	578	578	578	578	578	578	578
**R-squared**	0.940	0.940	0.939	0.939	0.945	0.945	0.952	0.952	0.943	0.958

Notes: The dependent variable is the modified RCA index based on the forward linkage-based measured of domestic value added. Standard errors in brackets. Robust standard errors clustered by country.

*p <0.05

**p < 0.01

***p < 0.001.

All variables are expressed in logs. All models include country and time fixed effects.

In column (2), we introduce the provider-weighted indicator of foreign services value added content of manufacturing exports (Eq 4). As expected, we obtain a positive and statistically significant coefficient for this variable, revealing that countries’ comparative advantage in manufacturing increases with foreign services intermediate inputs coming from more important suppliers of those services. This result provides strong support to our hypothesis: it is the level of expertise of foreign providers rather than the mere quantity of foreign services value added content of manufacturing exports that is relevant for manufacturing competitiveness.

Results from estimating the specification in Eqs (5) and (6), where we add the control variables (*Z*_*it*_), are reported in columns (3) and (4), respectively. The first one includes the unweighted *FSVA*_*o*_*inX*^*M*^_*i*_ and the second one the weighted one. It is relevant to note that by doing so we lose many observations because of missing values for some countries and years. We observe that, in these estimations, both the unweighted and the weighted share of embodied foreign services value added are statistically significant in explaining manufacturing competitiveness with a positive effect. That is, both how much foreign services value added content and who the providers of those services are matter. The control variables included in the estimations show the expected signs. Labour productivity and size of the exporting country’s manufacturing sector are positively related to competitiveness. The effect of the country’s development level on comparative advantage in manufacturing is also positive although it is not statistically significant.

In columns (5) and (6), we add the hub score of the exporting country whose manufactures embody the foreign services value added (specifications in Eqs 7 and 8). The estimations results show that the higher the hub score of a country is, the higher its manufacturing competitiveness is. That is, being a central provider in the embodied services value added network positively affects the comparative advantage in manufacturing. Additionally, both the unweighted (column 5) and the provider-weighted (column 6) *FSVA*_*o*_*inX*^*M*^_*it*_ remain positive and statistically significant.

In columns (7) and (8), we add the share of domestic services value added embodied in a country’s manufacturing exports (*DSVAinX*^*M*^_*it*_) as an explanatory variable (specifications in Eqs 9 and 10). It displays a negative and statistically significant coefficient. That is, countries with higher domestic services value added content in their manufacturing exports exhibit relatively less comparative advantage in manufacturing. A possible explanation for this result is that manufacturing sectors that use domestic services in relatively high proportion might not be incorporating the highest specialised (expert) services available in the world market. In these estimations, both unweighted (column 7) and weighted (column 8) indicators of embodied foreign intermediate services lose their statistical significance, although they remain positive. The high partial correlation between the domestic component and the (weighted and unweighted) foreign component of the services value added embodied in manufacturing exports (around 0.5) might explain that lack of statistical significance.

Columns (9) and (10) display the results when the combined variable of quantity of domestic intermediate services in manufacturing exports and exporting country hub score is included in the specifications (Eqs 11 and 12). The coefficient of this weighted *DSVAinX*^*M*^ variable is positive and statistically significant. That is, countries’ comparative advantage in manufacturing increases with embodied services value added coming from more expert domestic providers. A similar outcome (positive and statistically significant effect) is found for both the unweighted (column 9) and the provider-weighted (column 10) indicator of foreign services. Furthermore, the higher magnitude of the coefficients of those variables suggests a stronger effect on manufacturing competitiveness compared with that of the country-weighted indicator of domestic services.

We go one step further and divide embodied services value added into types of services. As the share of value added content from different services categories is highly correlated among these categories, we estimate the effect of each category of services separately. Estimation results are reported in Table 4. Columns (a) and (c) display the results from estimating the specifications in Eqs (5) and (11), respectively, where the effect of unweighted *FSVA*_*o*_*inX*^*M*^ is estimated. The results which correspond to Eqs (6) and (12) are displayed in columns (b) and (d), respectively, where the impact of provider-weighted *FSVA*_*o*_*inX*^*M*^ is reported. In columns (c) and (d), the weighted *DVSAinX*^*M*^ is also estimated.

**Table 4:** Empirical model estimation results by types of services.

	**Wholesale and retail services**				**Transport and telecommunication services**			
**VARIABLES**	Column 1a	Column 1b	Column 1c	Column 1d	Column 2a	Column 2b	Column 2c	Column 2d
FVAS_o_inX^M^_it_	0.269***		0.179**		0.222***		0.139*	
	(0.0628)		(0.0809)		(0.0746)		(0.0723)	
o_weighted_FVAS_o_inX^M^_it_		0.225***		0.185***		0.232***		0.197***
		(0.0396)		(0.0480)		(0.0542)		(0.0473)
*i*_weighted_ DVASinX^M^_it_			0.0337**	0.0282**			0.0417***	0.0371***
			(0.0137)	(0.0113)			(0.0134)	(0.00756)
Employment	0.318***	0.305***	0.271**	0.265**	0.360***	0.353***	0.345***	0.342***
	(0.103)	(0.0991)	(0.106)	(0.101)	(0.103)	(0.0982)	(0.0970)	(0.0869)
Labour Productivity	0.0889**	0.0304	0.0571	0.0146	0.0963*	0.0783**	0.0590	0.0497
	(0.0409)	(0.0363)	(0.0424)	(0.0360)	(0.0478)	(0.0337)	(0.0511)	(0.0390)
GDPPC	0.150	0.121	0.0799	0.0763	0.131	0.143	0.0483	0.0785
	(0.0958)	(0.0870)	(0.116)	(0.0965)	(0.106)	(0.101)	(0.119)	(0.0911)
Observations	578	578	578	578	578	578	578	578
R-squared	0.944	0.949	0.946	0.951	0.941	0.949	0.946	0.953
	**Financial services**				**Computer, R&D and other business services**			
**VARIABLES**	Column 3a	Column 3b	Column 3c	Column 3d	Column 4a	Column 4b	Column 4c	Column 4d
FVAS_o_inX^M^_it_	0.119		0.0664		0.0853	**Col4c**	0.0395	
	(0.0731)		(0.0807)		(0.0697)		(0.0718)	
o_weighted_FVAS_o_inX^M^_it_		0.216***		0.197***		0.166**		0.132*
		(0.0560)		(0.0588)		(0.0673)		(0.0687)
*i*_weighted_ DVASinX^M^_it_			0.0249**	0.0182**			0.0291	0.0214
			(0.0099)	(0.0078)			(0.0181)	(0.0152)
Employment	0.343***	0.354***	0.348***	0.355***	0.364***	0.369***	0.341***	0.354***
	(0.107)	(0.101)	(0.101)	(0.0975)	(0.105)	(0.106)	(0.102)	(0.105)
Labour Productivity	0.0878**	0.0825**	0.0727*	0.0725***	0.0880*	0.0793*	0.0851*	0.0794*
	(0.0432)	(0.0341)	(0.0380)	(0.0261)	(0.0488)	(0.0454)	(0.0454)	(0.0432)
GDPPC	0.0791	0.149	0.0514	0.124	0.0669	0.0671	0.0215	0.0334
	(0.112)	(0.103)	(0.116)	(0.102)	(0.114)	(0.108)	(0.117)	(0.111)
Observations	578	578	578	578	578	578	578	578
R-squared	0.937	0.947	0.940	0.949	0.936	0.939	0.938	0.940

Notes: The dependent variable is the modified RCA index based on the forward linkage-based measured of domestic value added. Robust standard errors in brackets, clustered by country.

*p <0.05

**p < 0.01

***p < 0.001.

All variables are expressed in logs. All models include country and time fixed effects. To save space, constant terms are not reported.

For both *Wholesale and retail services* and *Transport and telecommunications services*, the share of embodied foreign services value added affects manufacturing competitiveness positively, and the effect is statistically significant (columns 1a and 2a). The effect is similar (positive and statistically significant) to that of the provider-weighted foreign services value added (columns 1b and 2b). That is, for these two services categories, which are considered traditional services, both how much foreign services content and who the providers of those services are matter.

However, for the other two services categories (*Financial services and Computer*, *R&D and other business services*), no statistically significant association between manufacturing RCA and embodied foreign services value added is observed (columns 3a and 4a). However, a positive and statistically significant association is found when the provider-weighted indicator of embodied foreign services value added is used (columns 3b and 4b). This result implies that a higher foreign services value added content from more central providers tends to increase manufacturing competitiveness. For these modern services, which are dynamic, internationally traded services and more complex services sectors [30, 31], the level of expertise of the provider (proxied by its hub score) emerges as a key factor for manufacturing competitiveness.

The last two columns for each services category (columns c and d) show that revealed comparative advantage in manufacturing increases with the share of domestic services value added weighted by the exporting country’s *hub* score. This happens for all the services types (columns 1c-1d, 2c-2d and 3c-3d), except for *Computer*, *R&D and other business services*, where the coefficient is not statistically significant (columns 4c-4d). Furthermore, in the estimations where the unweighted indicator of embodied foreign services value added is included (columns c), this variable positively and significantly affects manufacturing competitiveness only when the embodied services are *Wholesale and retail services* and *Transport and telecommunications services*. However, for the four types of services, the provider-weighted indicator of *FSVA*_*o*_*inX*^*M*^ shows a positive and statistically significant coefficient (columns d), although the magnitude of its effect on manufacturing RCA is slightly lower compared with that of previous estimates. That is, again the level of expertise of the services suppliers more than the quantity of those embodied services is relevant for manufacturing competitiveness in the case of modern services. From these estimations, an additional conclusion can be drawn: except for the last type of service, *Computer*, *R&D and other business services*, improvements in manufacturing competitiveness are associated with higher shares of both foreign and domestic services content of manufacturing exports when the centrality of the country provider is taken into account.

The different behaviour of the last services category may be related to the fact that they are knowledge-intensive services and hence more exclusive. Not all manufacturing industries incorporate *Computer*, *R&D and other business services* with the same intensity. The use of these modern services is more intensive in manufactures with a higher technological content. Consequently, only in manufacturing sectors with a high degree of embodied business services, would improvements in manufacturing competitiveness be associated with higher intermediate services provided by suppliers that are more expert. In fact, in Section 3.2, we detected that this sectional network is the least connected and integrated, the most diversified and the one in which the contribution of each country’s exchanged flows has diminished to the highest extent.

Therefore, our findings reveal the importance of the source country of foreign intermediate services in manufacturing exports for manufacturing competitiveness. While previous literature has focused on the quantity of foreign services inputs, according to the estimations above, the centrality of the country provider of those embodied foreign services inputs is also a key determining factor for improvements in manufacturing RCA.

## 5. Final considerations

In this paper we have analysed the importance of the source-country of foreign services value added embodied in manufacturing exports for manufacturing competitiveness. In order to do this, we have examined the characteristics of the inter-sectoral global network formed by flows of embodied services value added, paying special attention to the role that countries play within that network.

We have observed that the network exhibits a low density, with a connectivity that could be considered incipient as the number of countries that do not participate within the network in a significant way is still relatively high. The structure of the network is regular (not a centre-periphery) with a numerous group of highly connected countries in the centre with trade relationships with many other countries. New countries have been incorporated into this centre over time, playing a prominent role: China is the most evident case. Other countries, still poorly connected, are becoming incorporated into the network little by little, spreading it out. We have also detected that the most connected and integrated countries are also the most intensely connected, and that those countries are the ones which have strengthened their links over time. Therefore, the analysis suggests that the network can still become more developed, connected and cohesive in the future and that those central countries within the network will be the ones that bolster their future integration.

Three relevant centres have been detected within the network, with a regional scope and connected themselves: the European Factory, with the central EU economies as leaders, particularly with Germany at the forefront; the North American Factory, with the United States as the principal country; and the Asian Factory, with China at the head. Within those three centres, there are both “expert suppliers” and “expert recipients”, meaning countries that supply embodied services value added not only to other economies which do not produce them themselves, but also to other countries which are significant providers, themselves central countries within the network as suppliers.

These hubs are able to provide highly specialised intermediate services to their partners, which, in turn, are sophisticated recipients themselves. In this regard, we have showed that for manufacturing competitiveness, together with the level of embodiment of services value added into manufacturing exports, expertise as providers of countries’ suppliers is also relevant: the higher the centrality of intermediate services suppliers, the more intense the competitiveness-enhancing effect is. Therefore, who the providers of those embodied services inputs are is also a key determining factor for manufacturing competitiveness.

These findings would suggest that, in the near future, this inter-sectoral network will continue to develop and widen, deepening the servicification of manufactures, especially those destined for exports. In this process, we have seen how the network of embodied services value added is increasingly incorporating new emerging economies, which are, hand in hand with GVCs of manufactures, becoming crucial actors within the inter-sectoral world network. Although the most advanced economies remain at the top of the hubs ranking, now and even more in the near future, they will have to share their positions with these new economies and others, which presumably will become integrated within the network, especially in those more traditional services. It seems undoubtable that in this process, the current central countries and the ones which will be incorporated into the network centre will be the driving forces of this process, playing a crucial role in the improvement of industry competitiveness. They will be the economies which will provide the global manufacturing sector with the most specialised and advanced services; in turn, this specialisation will imply that those same economies will also demand other sophisticated services from other partners. This interdependence will knit an increasingly denser and more integrated network.

## Supporting information

S1 Appendix (DOCX)
