# Supplementary material for: The role of services content for manufacturing competitiveness: A network analysis
Source: PLoS One. 2020 Jan 14;15(1):e0226411. doi: 10.1371/journal.pone.0226411 (PMC6959589; doi:10.1371/journal.pone.0226411)
Supplement: S1 Appendix — (DOCX) [file pone.0226411.s001.docx]

**Statistical Appendix**

**Table A.1. Countries list**

| Argentina (ARG) | Czech Republic (CZE) | Latvia (LVA) | Saudi Arabia (SAU) |
| --- | --- | --- | --- |
| Australia (AUS) | Denmark (DNK) | Lithuania (LTU) | Singapore (SGP) |
| Austria (AUT) | Estonia (EST) | Luxemburg (LUX) | Slovak Republic (SVK) |
| Belgium (BEL) | Finland (FIN) | Malaysia (MYS) | Slovenia (SVN) |
| Brazil (BRA) | France (FRA) | Malta (MLT) | South Africa (ZAF) |
| Brunei Darussalam (BRN) | Germany (DEU) | México (MEX) | South Korea (KOR) |
| Bulgaria (BGR) | Greece (GRC) | Morocco (MAR) | Spain (ESP) |
| Cambodia (KHM) | Hong Kong (HKG) | Netherlands (NDL) | Sweden (SWE) |
| Canada (CAN) | Hungary (HUN) | New Zealand (NZL) | Switzerland (CHE) |
| Chile (CHL) | India (IND) | Norway (NOR) | Thailand (THA) |
| China (CHN) | Indonesia (IDN) | Peru (PER) | Tunisia (TUN) |
| Chinese Taipei (TWN) | Ireland (IRL) | Philippines (PHL) | Turkey (TUR) |
| Colombia (COL) | Iceland (ISL) | Poland (POL) | United Kingdom (GBR) |
| Costa Rica (CRI) | Israel (ISR) | Portugal (PRT) | United States (USA) |
| Croatia (HRV) | Italy (ITA) | Romania (ROU) | Viet Nam (VNM) |
| Cyprus (CYP) | Japan (JPN) | Russian Federation (RUS) | Rest of the World (RoW) |

**Table A.2. Topological Measures of the (FISN) network, 1995 and 2011**

| Binary network | 1995 | 2011 |
| --- | --- | --- |
| First-order indicators |  |  |
| Arcs (#) | 558 | 615 |
| Density | 0.138 | 0.153 |
| Average Node Degree (average number of arcs) | 17.437 | 19.219 |
| Degree Centralization | 0.371 | 0.308 |
| *Indegree Centralization* | *0.456* | *0.555* |
| *Outdegree Centralization* | *0.520* | *0.474* |
| Closeness Centrality (Average) | 0.368 | 0.400 |
| Betweenness Centrality (Average)^a^ | 0.008 | 0.009 |
| Random Walk Betweenness Centrality (RWBC) | 0.137 | 0.141 |
| k-core | 17 (k=22) | 17 (k=23) |
| Clustering Coefficient^b^ | 0.626 | 0.611 |
| Second-order indicators |  |  |
| Average Nearest-Neighbor Degree (ANND) | 94.806 | 99.257 |
| Weigthed network | **1995** | **2011** |
| First-order indicators |  |  |
| Average Node Strength | 2.618 | 2.592 |
| Random Walk Weighted Betweenness Centrality (RWWBC) | 0.121 | 0.179 |
| Weighted Clustering Coefficient | 0.073 | 0.070 |
| Second-order indicators |  |  |
| Average Nearest-Neighbor Strength (ANNS) | 19.755 | 18.894 |

Source: Authors’ calculation based on OECD-OMC TiVA Database using the program package Pajek for analysis and visualization of large networks (<http://mrvar.fdv.uni-lj.si/pajek/>)

Notes: A detailed description of these topological measures can be found in the seminal book by Wasserman et al. [32]. ^a^ It measures the fraction of the shortest paths between pairs of nodes that go through the analysed node. In particular, we use in this study the *Random-walk betweenness centrality* index proposed by Newman [33] and Fisher and Vega-Redondo [34]. ^b^ It measures in which extent the partner of a country are also themselves partners and it calculates the number of triangles that are formed around a node, divided by the maximum number of triangles that could conform around it.

**Table A.3. Dependent and explanatory variables: definition and sources**

| **Variable** | **Definition** | **Source** |
| --- | --- | --- |
| **RCA^M^_DVAit_** | Ratio between the share of domestic manufacturing value added embodied in country *i*’ gross exports in the country’s total domestic value added embodied in gross exports at time *t*, and the corresponding share for the world. | OECD-WTO TiVA Database (December 2016) |
| **FSVA_o_inX^M^_it_** | Foreign services value added embodied in a country’s manufacturing exports as a share of those manufacturing exports |  |
| **o_weighted_FVAS_o_inX^M^_it_** | Provider-weighted indicator of foreign services value added content of manufacturing exports where the share of intermediate services from each supplier is weighted by each supplier’s hub score. |  |
| **DSVAinX^M^_it_** | Domestic services value added embodied in a country’s manufacturing exports as a share of those manufacturing exports. |  |
| **Hub_it_** | The value as a *hub* of the exporting country in the FISN |  |
| **i_weighted_DVASinX^M^_it_** | Country *i*-weighted indicator of domestic services value added content of manufacturing exports where the share of those domestic services is weighted by the exporting country’s hub score. |  |
| **Employment_it_** | Manufacturing employment in country *i*. | OECD Stan-Database |
| **Labour Productivity_it_** | Labour productivity in country *i* as real value added divided by total employment. |  |
| **GDPPC_it_** | Gross Domestic Product *per cápita* of country *i*. | World Development Indicators. The World Bank. |

**Table A.4. Some descriptive statistics of the model variables**

| **Variable** | **Observations** | **Mean** | **Std. Dev.** | **Min.** | **Max.** |
| --- | --- | --- | --- | --- | --- |
| RCA^M^_DVAit_ | 1,088 | 0.896 | 0.310 | 0.019 | 1.736 |
| FSVA_o_inX^M^_it_ | 1,088 | 15.489 | 6.590 | 3.11 | 40.78 |
| o_weighted_FVAS_o_inX^M^_it_ | 1,088 | 19.255 | 7.077 | 2.24 | 43.14 |
| DSVAinX^M^_it_ | 1,088 | 0.065 | 0.106 | 0 | 0.794 |
| Hub_it_ | 1,088 | 1.261 | 1.921 | 0 | 10.545 |
| i_weighted_DVASinX^M^_it_ | 1,088 | 2.809 | 1.393 | 0.652 | 8.481 |
| Employment_it_ | 598 | 2197.399 | 3390.961 | 17.4 | 18222 |
| Labour Productivity_it_ | 578 | 63.919 | 39.963 | 7.438 | 277.448 |
| GDPPC_it_ | 1,037 | 28233.47 | 17874.25 | 2142.728 | 107008.1 |
